# An analysis on the electrophoretic mobility of cellulose nanocrystals as thin cylinders: relaxation and end effect†

**DOI:** 10.1039/c9ra05156b

**Published:** 2019-10-22

**Authors:** Kuan-Hsuan Lin, Donghao Hu, Takuya Sugimoto, Feng-Cheng Chang, Motoyoshi Kobayashi, Toshiharu Enomae

**Affiliations:** Graduate School of Life and Environmental Sciences, University of Tsukuba 1-1-1 Tennodai Tsukuba Ibaraki 305-8572 Japan enomae.toshiharu.fw@u.tsukuba.ac.jp; Graduate School of Agricultural and Life Sciences, The University of Tokyo 1-1-1 Yayoi, Bunkyo-ku Tokyo 113-8657 Japan; School of Forestry and Resource Conservation, Advanced Research Center for Green Materials Science and Technology, National Taiwan University No. 1, Sec. 4, Roosevelt Rd. Taipei 10617 Taiwan

## Abstract

Cellulose nanocrystals (CNCs) are extracted from cellulosic fibers *via* sulfuric acid hydrolysis and found to exhibit unique properties due to their nanoscale, ordered structure, and surface morphology. The dispersion stability of a CNC suspension is a significant factor when CNCs are applied for reinforcement of a composite or ink jet printing. Since sulfuric acid hydrolysis introduces sulfate groups on CNC surfaces, we considered that charging conditions needed to be characterized, typically based on electrophoretic mobility. After the electrophoretic mobility was measured, several theoretical equations were applied to fit those values to assume the proper CNC particle shape. While Smoluchowski's equation is often used for this purpose, its applicability to CNCs should be reconsidered due to the thin, rod-like shape of CNCs with a finite length and high charge density. In this sense, we measured the surface charge and electrophoretic mobility of well-characterized CNCs. The obtained experimental data have been analyzed by using various electrokinetic equations. Our analytical results suggested that Smoluchowski's equation and the Ohshima–Henry equation overestimated the magnitude of the mobility of CNCs because it ignores the double layer relaxation and end effect. They also suggested that neither the Ohshima–Overbeek averaged equation nor the Ohshima–Overbeek perpendicular equation described the mobility of CNCs appropriately because those equations consider the double layer relaxation and end effect of a cylinder in a limited manner. Instead, the modified Ohshima–Overbeek equation was presented to be preferred for such a charged cylinder with a small aspect ratio.

## Abstract 2

Cellulose nanocrystals (CNCs) are extracted from cellulosic fibers *via* sulfuric acid hydrolysis and found to exhibit unique properties due to their nanoscale, ordered structure, and surface morphology.

## Graphical abstract

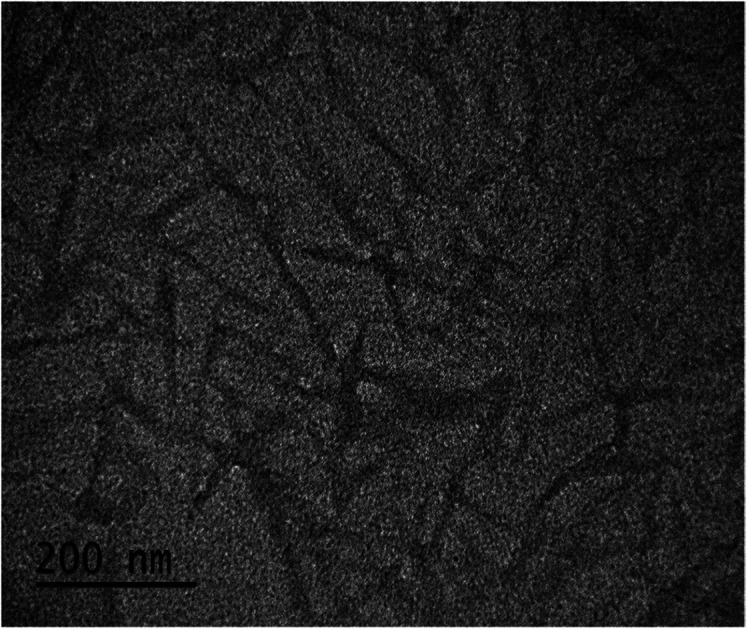

## Introduction

Cellulose is a sustainable bioresource and abundant in nature, and its chains are arranged in a highly ordered structure. Cellulose forms two regions: crystalline—called microfibrils—and amorphous regions, depending on the bonding mode of hydroxyl groups. With the nanometric size effect, cellulose nanocrystals (CNCs) extracted from crystalline regions have recently been studied in novel applications, such as cellulose biosensors,^1^ in mechanical property reinforcement,^2^ and in tissue engineering.^3^ Due to the steric effect, a hydroxyl group at the C6 position of a cellulose chain exhibits the highest reactivity. CNCs prepared *via* sulphuric acid hydrolysis are suggested to graft sulfate groups at the C6 position, making rod-like CNC particles well dispersed in an aqueous system due to repulsive forces.^4^ Thus, the understanding of the surface potential (*Ψ*) due to the charging of sulfate groups on the surface and an electrical double layer surrounding a CNC particle has become important to evaluate the stability of CNC suspensions.

Electric potential decreases with an increasing distance from the particle surface in an electric double layer. Zeta potential (*ζ*), defined as the potential at the slipping plane in an interfacial double layer, can be used to evaluate the level of dispersion in a colloidal suspension; this is usually calculated considering the present electrolytes, solvent and other suspended particles, and temperature, among other factors. Several equations have been formulated to obtain *ζ* from electrophoretic mobility (*μ*) because a value of *μ* can be easily obtained as the ratio of the velocity of particles to the applied electric field. For this purpose, Smoluchowski's equation (eqn (1)), which is suitable for large, spherical particles with a low *ζ*, was widely used in previous studies.^5,6^1
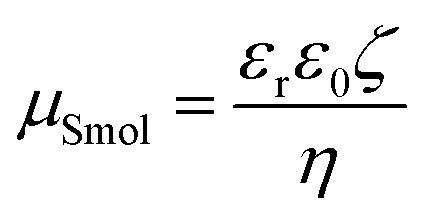
where *η* is the solvent viscosity, *ε*_0_ and *ε*_r_ are the dielectric constants of vacuum and water, respectively, and *ζ* is the zeta potential. However, CNCs are nanoscale thin rod-like particles with a *ζ* of approximately −25 mV (ref. 6) or with a surface charge density of approximately −0.1 C m^−2^.^7^ When the surface charge is high and the size is small, Smoluchowski's equation is no longer available because of retardation and relaxation effects. The retardation and relaxation effects can be accounted for by using the Henry equation and O'Brien-White program/Ohshima's equation for a sphere.^8,9^

For the case of cylindrical particles, Ohshima^10^ derived Henry's type approximate equation, eqn (2), for the electrophoretic mobility of a cylinder oriented perpendicular to an applied electric field *μ*^OH^_⊥_ and further suggested the use of an orientation-averaged equation *μ*_av_ by applying Smoluchowski's equation to an infinitely long cylinder oriented parallel to the applied electric field *μ*_‖_ = *μ*_Smol_ as eqn (3).2
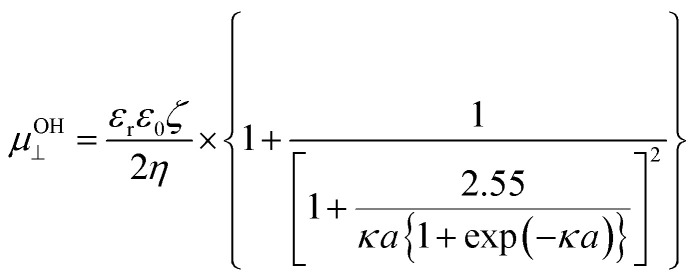
3
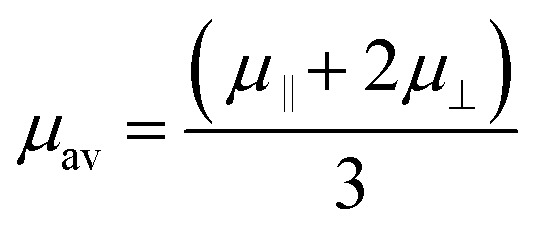


In eqn (2), *κ*, the Debye–Hückel parameter is introduced. The Debye length—thickness of double layer—was defined as an inverse of the Debye–Hückel parameter (*κ*^−1^). The theoretical relationship between *κ*^−1^ and the concentration of the added 1–1 electrolyte is defined as eqn (4)^11^4
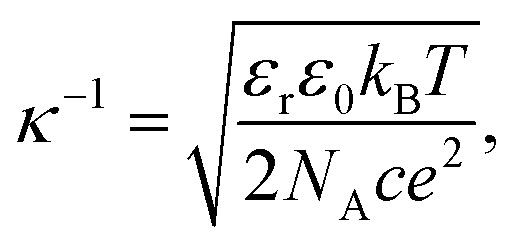
where *ε*_0_ and *ε*_r_ are the dielectric constants of water and vacuum, respectively, *k*_B_ is the Boltzmann constant, *T* is the absolute temperature, *N*_A_ is the Avogadro number, *e* is the elementary charge, and *c* is the concentration of 1–1 electrolyte. Therefore, the product of *κ* and the particle radius (*a*), *κa*, could be regarded as the ratio of the particle radius to the Debye length, which is related to the contribution of curvature due to the thin cylindrical shape and plays a critical role in colloidal systems. An averaged mobility of randomly oriented cylindrical particles is given by a suitable ratio of the mobility of cylinders that are migrated perpendicular and parallel to the electric field (eqn (3)).^12^ In this study, “Ohshima–Henry equation” was used to show the results calculated by Smoluchowski's equation and Henry's equation, respectively, and referred to as “*μ*^OH^_av_”.

Since *ζ* is an important parameter when evaluating the stability of CNC suspensions in aqueous media, it was applied to estimate the influence of counterion adsorption,^13,14^ raw material of CNCs,^15^ ultrasonic treatment on CNC suspensions,^16^ and the duration of acid hydrolysis in the CNC preparation process.^17^ However, the equation to derive *ζ* from *μ* is not detailed in either case,^13,15^ and Smoluchowski's equation is simply selected.^14,16^ The cylindrical shape of CNCs should be considered, and the Ohshima–Henry equation is used sometimes.^13,17^ Nevertheless, the effect of surface potential contributed by sulfate groups and the shape of a short cylinder also plays a role in the result of *μ*.

Therefore, when the number of sulfate groups present on the surface of a CNC introduced by sulphuric acid hydrolysis is significant, the high surface potential and the resulting relaxation effect need to be accounted for in the calculation. Ohshima (2015) derived equations with a consideration of the relaxation effect based on equations provided by van der Drift *et al.* (1979), which account for cases of high surface potential.^18^ With consideration of the valence of counterions (*Z*) and dimensionless ionic drag coefficient (*m*±), eqn (5) is the *μ* for a cylinder in the perpendicular orientation with the relaxation of the double layer (named “Ohshima–Overbeek perpendicular equation”). The averaged *μ*, “Ohshima–Overbeek averaged equation”, is represented by 
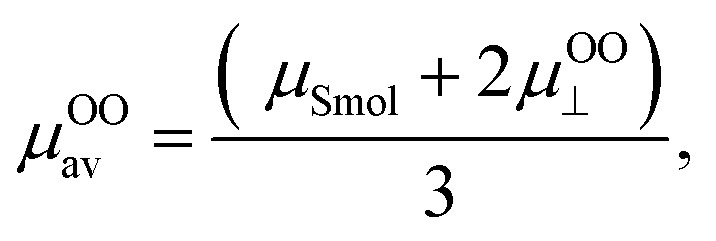
 according to Ohshima (2015). These equations are applicable to low to moderately high *ζ* (<100 mV).5
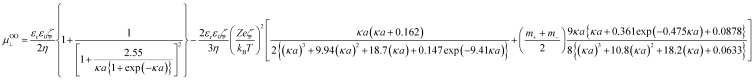


Eqn (1)–(5) consider different aspects of the properties of the particles such as cylindrical shape, surface potential, and the relaxation effect and thus are suitable for estimating the charging properties of CNC colloidal particles. Nevertheless, to the best of the authors' knowledge, the application of eqn (1)–(5) to CNC has never been examined. The objective of this study was to examine the applicability of the theoretical equations to the electrophoretic mobility of well-characterized CNC in terms of the shape and surface charge.

## Experimental

### Sample preparation

Commercially available CNCs purchased from University of Maine, USA, were used. Suspension samples were prepared as follows: the purchased freeze-dried CNC powder was dispersed in distilled water to a concentration of 0.1% (w/w); 1 mM HCl or NaOH was added to it to adjust the suspension pH to 3, 5, 7, 9, and 11. After preparing CNC suspensions with the specified pH values, different amounts of NaCl were added to each of them to evaluate an electrolyte addition effect, and the concentration was adjusted to 1, 3, 5, 10, 15, 20, 25, 30, 35, 40, 45, and 50 mM. Some samples were also prepared without any acid or base for pH adjustment except fresh distilled water.

### Dimensional measurements

Two methods were applied for determining dimensional properties. Transmission electron microscopy (TEM) (JEM-1200 EXII, JEOL, Japan and H7650, Hitachi, Japan) was used to measure the dimension of CNCs after drying on a TEM grid. A hydrodynamic size distribution was measured based on the dynamic light scattering technique. For TEM measurements, CNC suspensions at 0.1% (w/w) were dropped on 20 mesh carbon-coated TEM copper grids for TEM observations at 60 kV in accelerating voltage. The particle dimensions were analysed using ImageJ (https://imagej.nih.gov/ij/index.html) for at least 30 different particles. Only individual particles with clear edges were measured. The length and width were determined as the distance between the two ends of each particle along the length axis and distance between the edges across the axial centre, respectively.

The Stokes–Einstein equation introduces a relationship between the diffusion coefficient by Brownian motion. Then, the particle size and hydrodynamic size distribution of CNC particles were determined from the diffusion coefficient of particles indicated by the rate of fluctuation of the signal intensity analysed by autocorrelation built in the instrument (Zetasizer Nano-ZS, Malvern Instruments, England).

### Potentiometric titration

The surface charge of CNCs was measured by potentiometric acid–base titration with a pH meter integrated with a burette (Easy pH, Mettler Toledo, USA). A blank solution was prepared by mixing 25 mL of 0.01 M NaCl with 10 mL of 0.01 M HCl. Then, a titration curve of the blank was constructed by using 0.01 M NaOH under a CO_2_-free system with continuous stirring and bubbling argon gas. A CNC suspension at approximately 0.3% (w/w) with 0.01 M HCl was then added into the blank sample and titrated again. Titration curves of the blank and CNCs were plotted with pH values calibrated by Gran's plot. The surface charge density (*σ*) was calculated from the volumes of the titrant added to blank and the suspension of CNCs at the same pH, CNC density at 1.6 g cm^−3^,^19^ and the specific surface area.

### Elemental analysis

Contents of N, C, S, and H were determined by elemental analysis (Elemental analyser: elementar vario EL cube, Germany). CNCs were packed into a tin-capsule and incinerated at a higher temperature than 1000 °C in the presence of oxygen to obtain oxides of N, C, and S. Subsequently, the mass of the oxides was detected and converted to the composition.

### Zeta potential calculation

If the surface charge density is constant irrespective of pH or electrolyte concentration as expected from sulphate groups on CNCs, one can evaluate the surface potential as a function of the electrolyte concentration by using the solution following the Poisson–Boltzmann (PB) equation (Fig. S1 in the ESI†). *ζ*_theoretical_ of CNCs in the 1–1 electrolyte solution was estimated by solving the PB equation as an electric potential at the slipping plane in an electrical double layer with the idea reported by Ohshima (1998).^20^ The relationship between the surface charge density, *σ*, and the surface potential, *ψ*_s_, is approximated as eqn (6)–(8):6
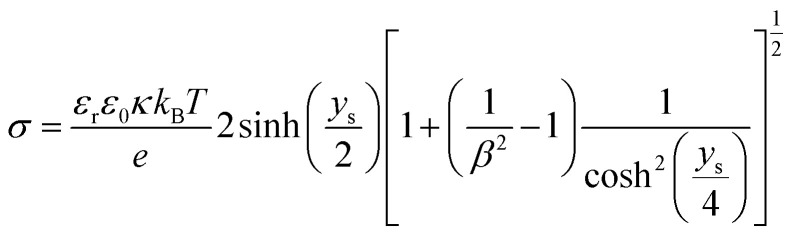
7
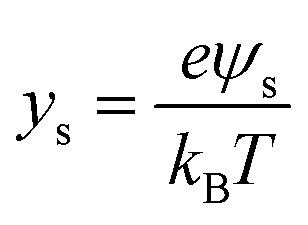
8
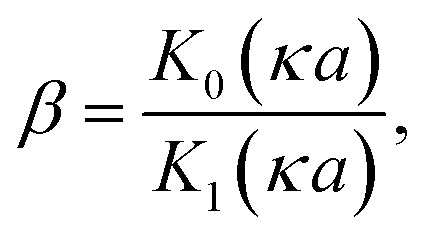
where *K*_*n*_(*x*) is the modified Bessel function of the second kind of order *n*.

Then, when the distance from the surface of a cylinder to an assumed slipping plane is decided as *X*_s_, *ψ*(*X*_s_) could be written as9
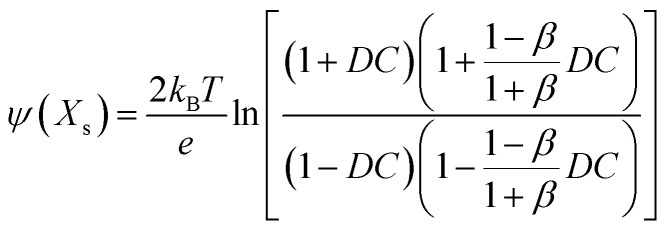
where10
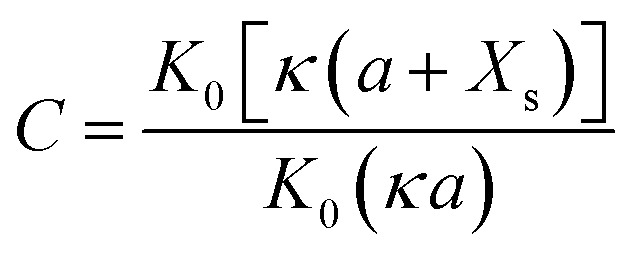
and11
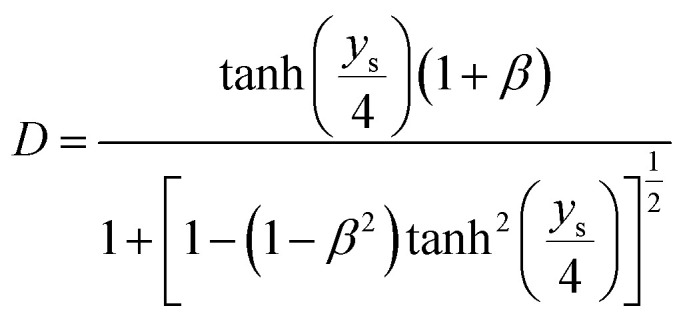


Thus, *ζ*_theoretical_ could be approximated as the surface potential located at *X*_s_ from the particle surface.

### Zeta potential and electrophoretic mobility measurements

Charged particles can electrophoretically migrate in solution in an applied electric field. The migration velocity of particles under these conditions is measured by laser Doppler velocimetry. The measured mobility (*μ*_measured_) was then calculated from a ratio of the migration velocity to the electric field intensity, reflecting the surface potential of colloidal particles. *ζ* of CNCs under different conditions were calculated based on the *μ*_measured_ using theoretical equations such as Smoluchowski's theory (eqn (1)). Solid polarization was reported to significantly affect electrophoretic mobility only when an applied electric field was strong and a dielectric permittivity ratio of particle to solution was large.^21^ In this study, however, the applied electric field was weak and the dielectric permittivity ratio was small. Thus, the solid polarization effect is negligible. Data of *μ*_measured_ were collected at 20 °C using a Zetasizer from 20 data points after 3 runs, and the conversion for *ζ*_Smol_ was based on Smoluchowski's theory.

## Results and discussion

### Dimensional information of dried CNCs

**Fig. 1:**
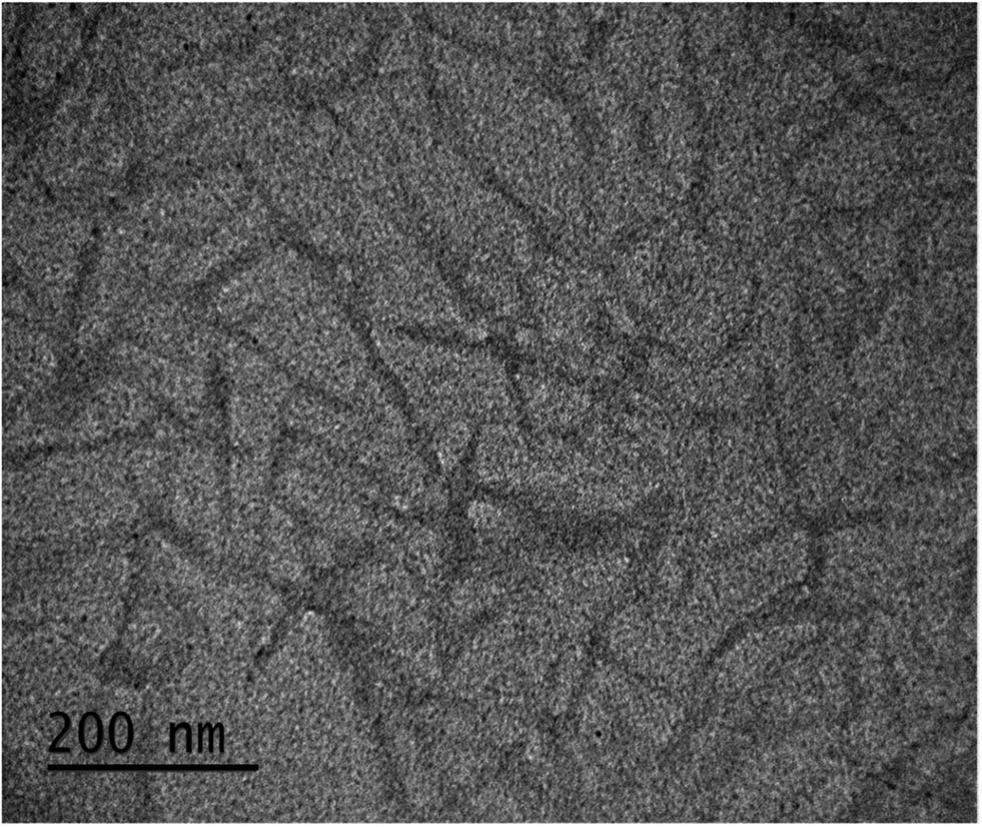
Transmission electron micrograph of CNCs.

Fig. 1 shows a TEM micrograph of CNCs with information about dimensions. Spindle-shaped CNC particles captured in this TEM image were analysed, and their average length (*L*) and width (*D*) were determined to be 168 ± 3 nm and 13.5 ± 0.5 nm, respectively. Based on this information, the aspect ratio of CNCs (*L*/*D*) was calculated to be approximately 12.4.

### Influence of pH and electrolyte concentration on electrophoretic mobility

**Fig. 2:**
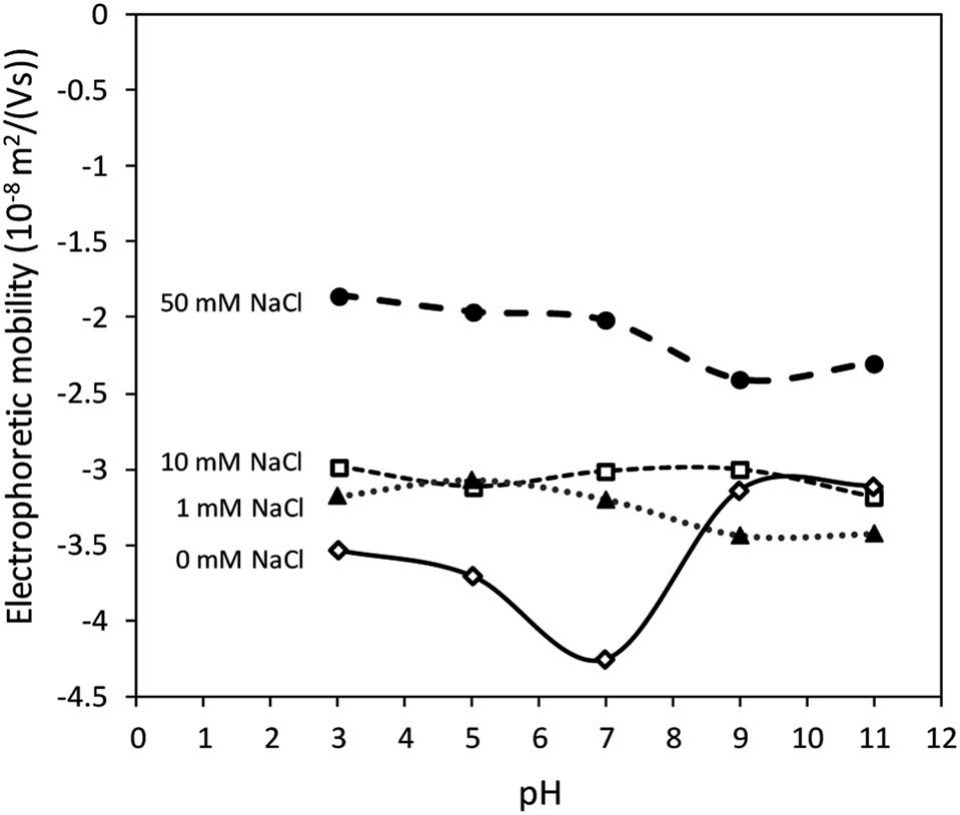
Relationship between electrolyte concentrations and *μ*_measured_ of CNCs at various pH values.

Fig. 2 shows that the electrolyte concentrations played a more important role in influencing the value of electrophoretic mobility than pH values. Sulphate groups (–OSO_3_^−^) were supposed to provide a pH-independent *μ*_measured_, as well as a *ζ* value on the surface of CNCs as mentioned in previous studies.^22–25^ The result demonstrates the constant surface charge density of the CNCs. The CNCs showed low electrophoretic mobility values obtained from the Zetasizer (*μ*_measured_) with additional electrolyte due to the compressed electric double layer. Although the surface charge was constant, the value of *μ*_measured_ of CNCs decreased with increasing salt concentration due to the compressed electric double layer.

### Charging behaviour and surface environment of CNCs

**Fig. 3:**
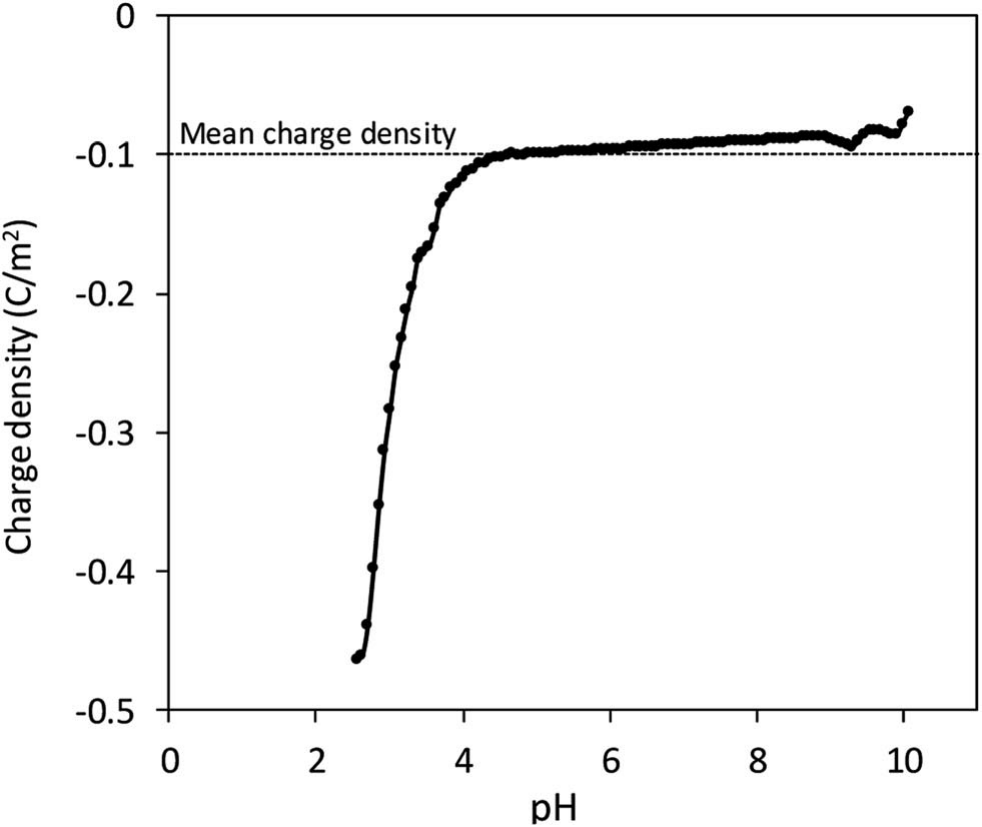
Surface charge density of CNCs.

We propose that sulphate groups dominate the colloidal behaviour of CNCs with a pH-independent *ζ* value as the previous result. Accordingly, potentiometric acid–base titration was applied to determine the charging behaviour, which might be influenced also by sulphate groups. Surface charge of CNCs resulted in a difference between titration curves of the CNC suspension and blank solution. The surface charge density was defined with the specific surface area of 304.2 m^2^ g^−1^ obtained by dimensional information from TEM and an assumed cylindrical shape (Fig. 1), with the assumption of a non-aggregated state. Fig. 3 shows that the surface charge density of CNCs was determined to be approximately −0.1 C m^−2^ after avoiding possible error from the strongly curved titration curve at low pH, which is close to that of sulphate group-grafted CNCs prepared *via* sulfuric acid hydrolysis.^7^ As shown in Table 1, the detection of S by elemental analysis also suggested the presence of sulphate groups on the surface of CNCs. By counting backward with the relationship between the surface charge density and sulfur content, entire dissociation of sulphate groups of CNCs were derived from approximately the same level of %S between the two methods.^26,27^

**Table 1:** Elemental composition of CNCs

Element	N	C	S	H
Ratio (wt%)	0.26	39.63	0.97	6.27

### Effect of electrolyte addition on the double layer of CNCs

**Fig. 4:**
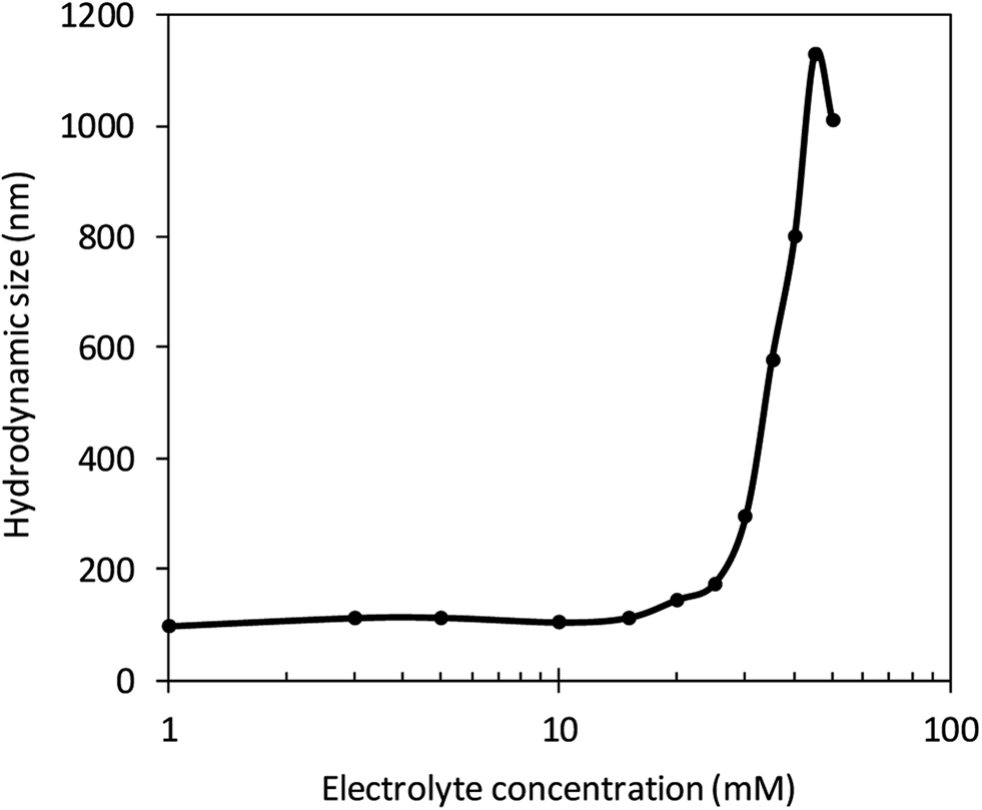
Relationship between electrolyte concentration and hydrodynamic size at different pH levels.

With more electrolyte addition, *κa* increased, meaning that the double layer became thinner and thinner. Fig. 4 shows that the hydrodynamic size was observed to increase quickly over an electrolyte concentration of 25 mM from experiment results. At this concentration, the hydrodynamic size was 173 nm and the *κa* value was 3.5. Thus, a CNC suspension with an electrolyte solution concentration higher than 25 mM showed sudden aggregation. With increased concentration from this level, the double layer became thinner and CNC colloidal particles became more aggregated.

### Analysis of electrophoretic mobility of CNC

**Fig. 5:**
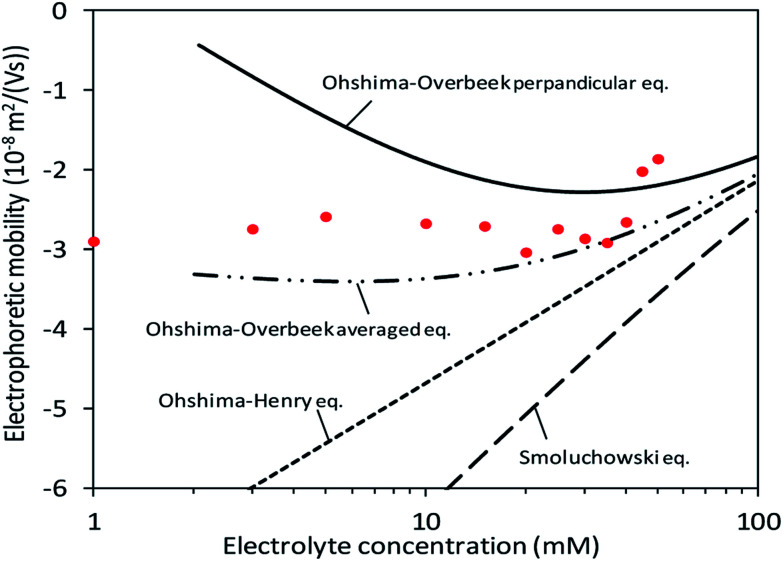
Fitting of theoretical equations when the surface charge density and distance between the particle surface and slipping plane are assumed to be −0.107 C m^−2^ and 0.65 nm, respectively.

In the calculation of electrophoretic mobility from theories (*μ*_theoretical_), it was assumed that the surface charge density was constantly −0.1 C m^−2^ as shown in Fig. 3 and previous studies^7,28,29^. Subsequently, the theories regarding orientation and the relaxation effect as well as the shape and surface potential of CNCs (eqn (1)–(5)) provided theoretical curves of *μ*_theoretical_. Fig. 5 exhibits those theoretical curves for fitting to the *μ*_measured_ on the condition of the slipping plane located 0.60–0.75 nm away from the particle surface.

At low electrolyte concentrations, the Ohshima–Overbeek averaged equation and Ohshima–Overbeek perpendicular equation curves fairly fitted the data points better than the others. This confirmed the assumption that CNCs have highly charged surfaces, indicating that the relaxation of the double layer reduces *μ*. To be more exact, however, neither the Ohshima–Overbeek averaged equation nor the Ohshima–Overbeek perpendicular equation fitted the data points, but the experimental data remained between both equations.

The Ohshima–Overbeek perpendicular equation did not fit the data points exactly due to the cylindrical shape of CNCs, suggesting a possible orientation effect. *μ*^OO^_av_ considers the orientation effect of CNCs, however, does not consider the end effect sufficiently. As mentioned before, *μ*^OO^_av_ uses *μ*^OO^_⊥_ as the perpendicular orientation with a relaxation of the double layer, but adopts *μ*_Smol_, in which the parallel part ignored the relaxation effect. This means that the Ohshima–Overbeek averaged equation is not completely suitable for cases with the significant end effect. The data points of *μ*_measured_ fell close to *μ*^OO^_av_ at higher electrolyte concentrations or at higher *κa* values. With a relatively small double layer and a *κa* value greater than 3.5, a trend similar to the theoretical prediction was shown in experimental data, which meant that the end effect became less pronounced. On the other hand, cellulose nanofibres are shaped as a long cylinder with relatively low *ζ*, if mechanically prepared, than CNCs and are reported to exhibit behaviour predicted by “Ohshima–Overbeek perpendicular equation”,^30^ whereas the relatively short length and high surface zeta potential characteristic of CNCs was responsible for the difference between *μ*_measured_ and *μ*^OO^_av_. In addition, a misinterpretation of the partial end effect consideration in the case of a relatively thick double layer when the same equations were applied was reported, and it was suggested that the end effect played a significant role in this situation.^11^ Thus, another estimation was suggested in this reference to deal with such a short cylinder with a thick double layer and a non-negligible end effect, all characteristic of CNCs.

**Fig. 6:**
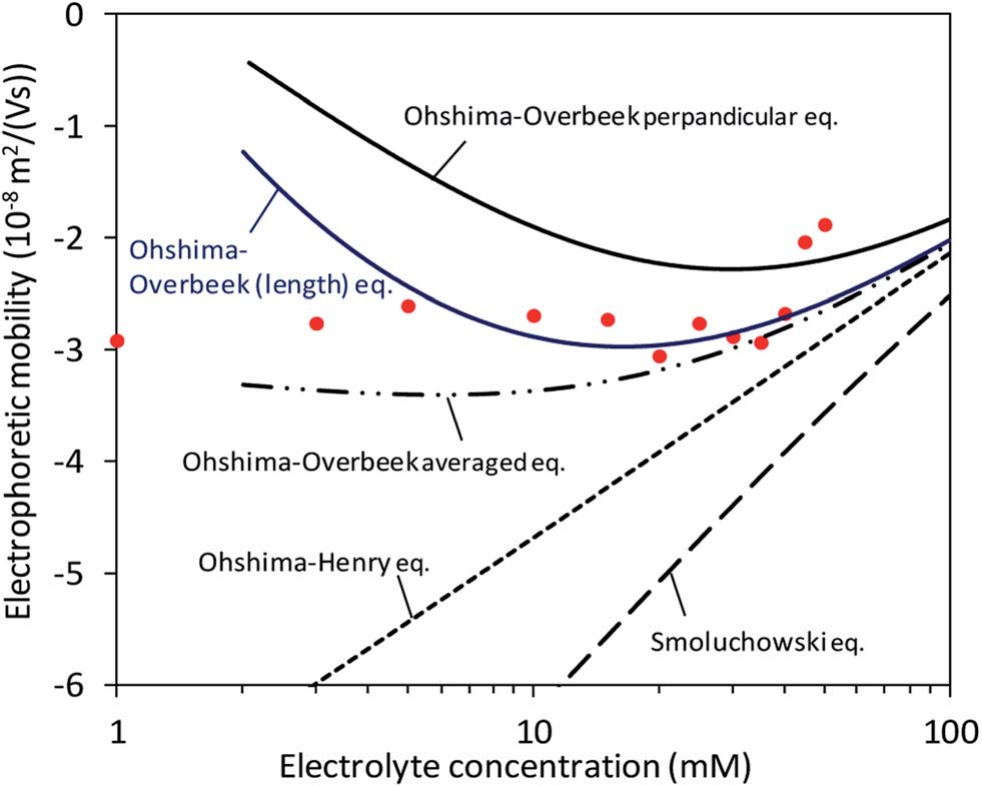
Fitting of theoretical equations including the Ohshima–Overbeek (length) equation as the best fitted approximation.

A concept to keep *μ*^OO^_⊥_ as *μ*^OO^_av_(*κa*) and replace *μ*_Smol_ with 
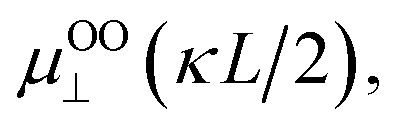
 where *L* is the length of the CNC particle, for a cylinder model oriented parallel to the field in calculating the averaged *μ* from Ohshima's equation (eqn (5)) proposed by Bakker *et al.*^11^ was applied. Then, a motivation of 
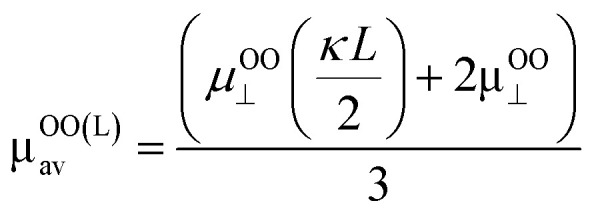
 was calculated and named as “Ohshima–Overbeek (length) equation”. Fig. 6 shows that this theory presented a much better fitting than Smoluchowski's theory for the parallel part. Nevertheless, the experimental data points were close to the Ohshima–Overbeek (length) equation, where the Ohshima equation was applied to both perpendicular and parallel parts for the cylinder mobility to account for the end effect. In addition, considering CNCs with various lengths, we confirm that “Ohshima–Overbeek (length) equation” becomes close to “Ohshima–Overbeek averaged equation” with increasing CNCs length (Fig. S2 in the ESI†).

Finally, our result suggests that the use of Smoluchowski's equation leads to the underestimation of *ζ* in magnitude. The relaxation effect and the end effect are crucial for parallel and perpendicular mobilities of CNCs due to high charge and a relatively small aspect ratio.

## Conclusions

A strongly negative surface potential was found in CNC suspensions when the relationship among pH, *ζ*, and electrolyte concentration was considered. Through the results of potentiometric titration and elemental analysis, sulphate groups introduced *via* sulfuric acid hydrolysis were proposed to possess such a strongly negative surface potential. The hydrodynamic size of CNC particles significantly increased at electrolyte concentrations higher than 25 mM, meaning that suspended CNC particles aggregated under this condition. Moreover, at a high electrolyte concentration, CNCs had a thin double layer with a high *κa* value, and thus the end effect was not very significant. At lower electrolyte concentrations (<25 mM), the double layer remained relatively thick (*κa* < 3.5) and the suspensions did not aggregate. In this situation, theoretical results such as the Ohshima–Overbeek averaged equation and the Ohshima–Overbeek perpendicular equation explained the experimental data more accurately than Smoluchowski's equation and Ohshima–Henry equation, owing to the cylindrical shape of CNC colloidal particles and the relaxation effect caused by the highly charged surface from sulphate groups. The reason that neither the Ohshima–Overbeek averaged equation nor the Ohshima–Overbeek perpendicular equation fitted the experimental data accurately might be the end effect, which became remarkable in the case of a short cylinder with a remarkable surface charge. Dealing with this problem, we successfully determined *μ* more exactly from *ζ* by a new method based on the Ohshima–Overbeek (length) equation that has never been applied to CNCs before.

## Conflicts of interest

There are no conflicts to declare.

## Supplementary Material

RA-009-C9RA05156B-s001
