# Supplementary material for: An analysis on the electrophoretic mobility of cellulose nanocrystals as thin cylinders: relaxation and end effect
Source: RSC Adv. 2019 Oct 22;9(58):34032–8. doi: 10.1039/c9ra05156b (PMC9073953; doi:10.1039/c9ra05156b)
Supplement: RA-009-C9RA05156B-s001 [file RA-009-C9RA05156B-s001.pdf]

Electronic Supplementary Material (ESI) for RSC Advances.
This journal is © The Royal Society of Chemistry 2019

SUPPROTING INFORMATION

An analysis on the electrophoretic mobility of cellulose nanocrystals as a thin cylinder: relaxation and end effect

Kuan-Hsuan Lin,*[a] Donghao Hu,[a] Takuya Sugimoto,[b] Feng-Cheng Chang,[c] Motoyoshi Kobayashi[a] and Toshiharu Enomae[a]

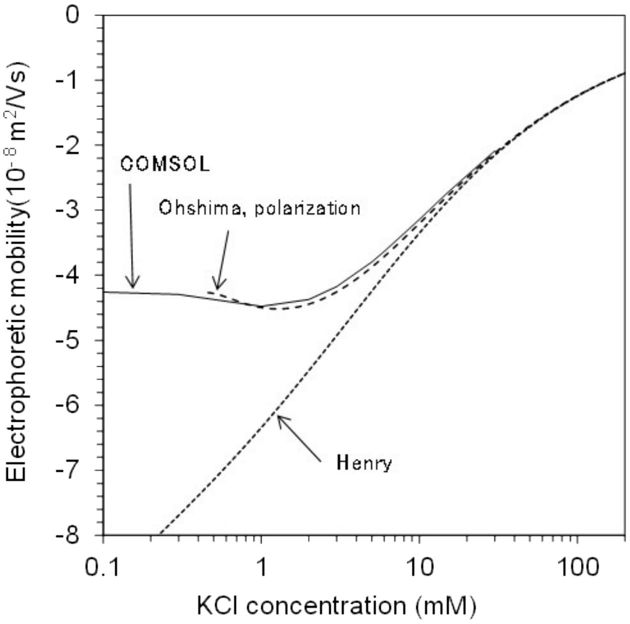

Figure. S1. When the Poisson-Boltzmann (PB) model is applied to obtain a zeta potential from surface charge density, the "Ohshima, polarization" eq. captures the double layer polarization effect (represented by a minimum mobility and reduction from the "Henry" eq.) and reasonably agrees with the numerical solution of Poisson-Nernst-Planck and the Stokes equations using "COMSOL", modelling software, for a sphere.

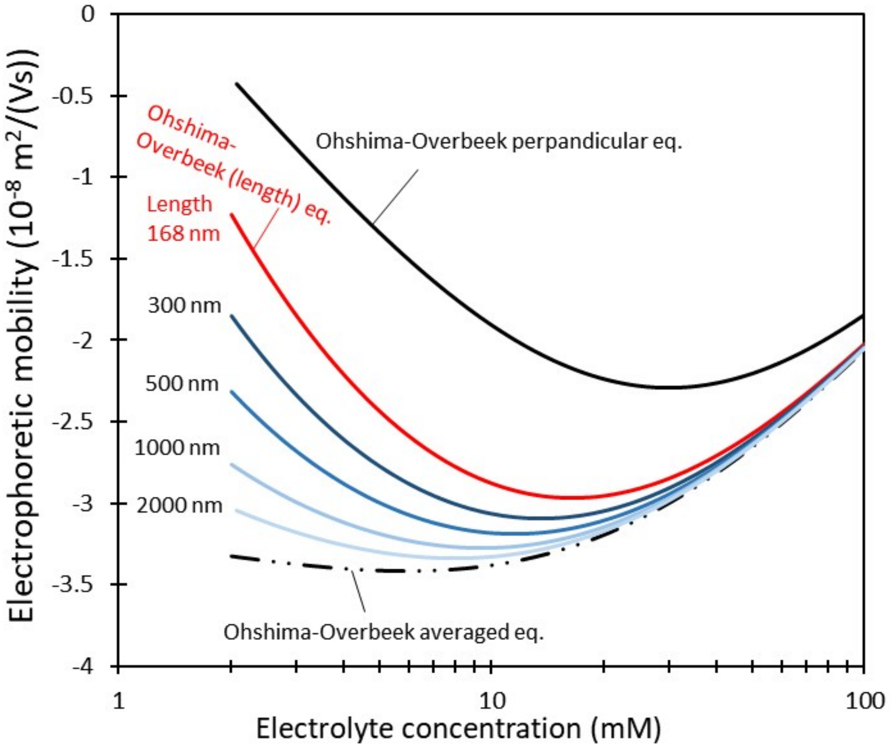

Figure. S2. An effect of the CNCs length depicted with "Ohshima-Overbeek (length) eq." with an assumption that the radius, zeta potential, and surface charge density are constant (independent with the length of CNCs). With increasing CNCs length, the "Ohshima-Overbeek (length) eq." curve becomes closer to "Ohshima-Overbeek averaged eq.", suggesting that the end effect plays a major role for shorter CNCs.
